# New Advances in π-Conjugated Materials

**DOI:** 10.3390/ma16186074

**Published:** 2023-09-05

**Authors:** Chuanqi Miao, Xiu Yu, Haichang Zhang

**Affiliations:** Key Laboratory of Rubber-Plastics of Ministry of Education/Shandong Province (QUST), School of Polymer Science & Engineering, Qingdao University of Science & Technology, 53-Zhengzhou Road, Qingdao 266042, China; 2021020070@mails.qust.edu.cn (C.M.); 13355423640@163.com (X.Y.)

Recently, extensive research efforts have been made to develop novel π-conjugated materials for use in various electronic applications, such as solar cells, organic semiconductors (OSCs), organic phototransistors (OPTs), organic light-emitting diodes (OLEDs), coatings, etc. These materials offer many technological advantages over their inorganic counterparts, such as solution processability, low fabrication cost, foldability, and easy conformation onto non-flat surfaces. In this specific research topic, two original works and a minireview have been reported, focusing on the applications of new π-conjugated materials. These studies provide a comprehensive exploration of different π-conjugated systems from various perspectives, with an emphasis on spectroscopic analysis.

With the rapid development of organic semiconductors (OSCs), the popularity of organic electrical components has increased rapidly. Compared with inorganic semiconductors, OSCs have the advantages of adjustable electronic properties, flexibility, low cost, versatility, and processability. However, most of the reported OSCs are hole-transport types (p-type), while fewer studies have been conducted on electron-transport (n-type) OSC materials. Indophenine dyes are a sub-family of quinoidal small molecules having an oxindole moiety as a terminal group, which has strong electron absorption ability and environmental stability. These excellent properties make indophenine dyes suitable for fabricating high-performance n-type materials; however, no articles on this topic has been published. 

Abderrahim Yassar’s group systematically summarized the recent state-of-the-art progress, while providing some guidelines for the design of quinoidal organic semiconducting materials [1]. At the same time, they discussed different synthetic strategies to improve the yield, reaction scale, regioselectivity, and product functionality of indocyanine dyes, which is significantly helpful for practical applications. However, there are still some problems that restrict the further development of indophenine-based materials. The indophenine reaction simultaneously produces quinoidal compounds of different bridging lengths and contains multiple isomers, which greatly limits the yield and purity of the material. This mixture of isomers is difficult to distinguish in NMR (nuclear magnetic resonance) spectra. To solve this problem, the authors propose engineering the π-bridge group, the intermolecular interactions can be used to drive the formation of one isomer. Moreover, the separation of materials with only one and two bridging groups is facilitated by exploiting the difference in solubility of small molecular materials of different lengths in various organic solvents. In addition, to improve the fabrication properties, engineering the side chains in the materials is a useful strategy. For example, introducing more soluble chains on oxindole rings facilitates the preparation of intrinsically highly soluble materials. In this way, the quinoloidal molecules are more soluble in non-halogenated organic solvents.

The use of C-H-activated cross-coupling and metal-free polymerization routes to OSCs are new research directions in this field. Furthermore, more in-depth research on the effects of various end-groups, π-conjugated cores, conjugated backbones, and side chains on the OFET performance is crucial, which are able to provide guides for synthesizing new generations of quinoidal-based OSCs with excellent performance, including tunable optoelectronic properties and more outstanding charge carrier mobility. 

Phototransistors are efficient devices that convert optical and electrical signals into each other. It has important applications in imaging system, communication technology, and environment and optical sensing. In order to prepare high-performance organic phototransistors (OPTs), ion dissociation and reducing dark current are the key factors. Reducing dark current can effectively reduce the noise of OPTs and improve the signal-to-noise ratio. Effective exciton dissociation is very important to maximize the generation of photogenerated carriers, so as to improve the optical response and performance of the whole device.

Lahane et al. used P3HT as the active layer to manufacture OPTs in a bottom gate top contact device architecture [2]. Small aluminum islands formed by floating film transfer method were observed in AFM images, and the number of aluminum islands increased with the increase in Al deposition thickness. XPS analysis showed that there was alumina on the surface of P3HT, and this alumina adsorbed and interacted with carbon (c) atoms in the edge-oriented P3HT alkyl chain to form Al-O-C bonds, but the strength of these bonds was weak and did not significantly affect the performance of the device. In addition, it was found that Al may bond with carbon molecules in P3HT, which is helpful in improving the performance. After changing the thickness of the deposited aluminum islands, the absorption peak of P3HT did not change significantly, but increasing the thickness of the aluminum islands led to light scattering. The polymer film deposited with the aluminum islands shows high crystallinity and good molecular order, which was confirmed by XRD and polarized absorption spectra. The results show that by using a unidirectional floating-film transfer method to deposit aluminum islands in the channel region, the channel width can be effectively reduced by depleting the P3HT layer, so as to reduce the dark current and promote the effective separation of excitons, and improve the device efficiency and optical response sensitivity. In addition, the effect of changing the thickness of Al islands on the properties of OPT was also investigated. The thickness of the Al islands was optimized to 5 nm. If the thickness of the Al islands exceeded 5 nm, the device encountered short circuit. Compared with the original device, the sensitivity of OPTs with a 5 nm aluminum island layer was significantly increased four-fold. The optimized device exhibited a saturated field-effect mobility of 2 × 10^−2^ cm^2^ V^−1^ s^−1^ and an impressive on–off ratio of 3 × 10^4^. It should be noted that these devices also show excellent photoelectric detection performance. Under the irradiation of 0.3 mW cm^−2^ and 525 nm, the photosensitivity was 2 × 10^5^, the optical responsivity was 339 AW^−1^, and the optical detection rate was 3 × 10^14^ Jones, EQE = 7.8 × 10^3^%. This work shows that reducing the depletion channel width of OPTs along with optimized metal islands can be used to optimize the performance and function of the device.

Skhirtladze synthesized two molecules by Buchwald–Hartwig coupling reactions with pyridazine as the electrophoretic core and 9,9-dimethyl-9,10-dihydroacridine or phenoxazine as the donor, which were called 2PO-PYD and 2AC-PYD, respectively [3]. They have high photoluminescence quantum efficiency, and the gap between the first excited singlet state (S1) to the first excited triplet state (T1) is small; this can contribute to efficient transfer from S1 to T1 by thermally activated delayed fluorescence (TADF).

From the synthesis perspective, Buchwald–Hartwig coupling reactions are mild and allow for optimal yields as well as production. In terms of thermal properties, 2PO-PYD and 2AC-PYD are both extremely thermally stable, with high heat loss and glass transition temperatures, and will not degrade with heat, but will sublimate directly. Regarding their geometries and electronic structures, the pyridazine molecules in 2PO-PYD and 2AC-PYD are bonded to the donor molecules in an almost perpendicularly crossed structure, which can provide a large space between donor and acceptor units and decrease the conjugation between the molecules; this is another reason why internal electric charges transfer from S0 to S1. Moreover, the gap between S1 and T1 is very small, which also lays the foundation for realizing efficient TADF. In terms of photophysical properties, 2PO-PYD and 2AC-PYD have excellent molecular energy levels; this was demonstrated by scanning their ICT bands which showed that both molecules are efficiently transferred from S1 to T1 by the TADF pathway. This work achieved efficient transferring from S1 and T1 by TADF, and it provides a reliable way for fluorescence and phosphorescence targeting control.
